# Association of Vitamin D Receptor Gene Variation With Osteoporosis Risk in Belarusian and Lithuanian Postmenopausal Women

**DOI:** 10.3389/fendo.2018.00305

**Published:** 2018-06-05

**Authors:** Pavel M. Marozik, Marija Tamulaitiene, Ema Rudenka, Vidmantas Alekna, Irma Mosse, Alena Rudenka, Volha Samokhovec, Katsiaryna Kobets

**Affiliations:** ^1^Laboratory of Human Genetics, Institute of Genetics and Cytology of the National Academy of Sciences of Belarus, Minsk, Belarus; ^2^Faculty of Medicine, Vilnius University, Vilnius, Lithuania; ^3^Department of Cardiology and Internal Diseases, Belarusian State Medical University, Minsk, Belarus; ^4^Department of Cardiology and Rheumatology, Belarusian Medical Academy of Postgraduate Education, Minsk, Belarus; ^5^Minsk City Center for Osteoporosis and Bone-Muscular Diseases Prevention, Minsk City Clinical Hospital, Minsk, Belarus

**Keywords:** vitamin D receptor, genetic variants, polymorphism, haplotype, postmenopausal osteoporosis

## Abstract

Vitamin D receptor (VDR) is one of the main mediators of vitamin D biological activity. VDR dysfunction might substantially contribute to development of postmenopausal osteoporosis (PMO). Numerous studies have revealed the effects of several *VDR* gene variants on osteoporosis risk, although significant variation in different ethnicities have been suggested. The main purpose of this work was to assess the frequency of distribution of *VDR* genetic variants with established effect and evaluate their haplotype association with the risk of PMO in a cohort of Belarusian and Lithuanian women. Case group included women with PMO (*n* = 149), the control group comprised women with normal bone mineral density (BMD) and without previous fragility fractures (*n* = 172). Both groups were matched for age, height, sex, and BMI—no statistically significant differences observed. *VDR* gene polymorphic variants (ApaI rs7975232, BsmI rs1544410, TaqI rs731236, and Cdx2 rs11568820) were determined using polymerase chain reaction and restriction fragment length polymorphism. The lumbar spine (L1-L4) and femoral neck BMD was measured using dual-energy X-ray absorptiometry. Association between each *VDR* variant and PMO risk was assessed using multiple logistic regression. The genotyping revealed statistically significant difference in the rs7975232 genotype frequencies between the patients and the controls (homozygous C/C genotype was overrepresented in patients, *p* = 0.008). Patients with osteoporosis were also three times more likely to carry the rs1544410 G/G genotype, when compared to controls. We found that rs7975232, rs1544410, and rs731236 variants were in a strong direct linkage disequilibrium (*p* < 0.0001), suggesting that risk alleles of these markers are preferably inherited jointly. For the bearers of C-G-C haplotype (consisting of rs7975232, rs1544410, and rs731236 unfavorable alleles), the risk of PMO was significantly higher (OR = 4.7, 95% CI 2.8–8.1, *p* < 0.0001) compared to controls. This haplotype was significantly over-represented in PMO group compared to all other haplotypes. Our findings highlight the importance of identified haplotypes of *VDR* gene variants. Complex screening of these genetic markers can be used to implement personalized clinical approach for prevention, treatment, and rehabilitation programs.

## Introduction

Vitamin D and its active metabolites are important components of the immune and hormonal systems that not only control phosphorus and calcium homeostasis but also play important role in providing numerous biological effects, involved in the regulation of processes of cell differentiation and proliferation in many target organs and tissues. So-called classical effects of 1.25(OH)_2_D and other vitamin D metabolites are participated in the processes of mineralization of bone tissue, maintaining calcium homeostasis and, finally, direct effect on bone remodeling mediated through the vitamin D receptor (VDR), encoded by *VDR* gene. Research published during the past two decades has established that pleiotropic effects of vitamin D and their genetic revelations are associated with a wide variety of diseases (1). This study is focused on association of *VDR* gene variants with susceptibility to postmenopausal osteoporosis (PMO).

Osteoporosis is characterized by reduced bone mineral density (BMD) and increased bone fragility. Homeostasis of bone tissue during lifetime is mainly maintained by balanced processes of bone resorption and formation, resulting from the combined action of multiple genes and environmental factors. Identification of gene variants, responsible for low BMD, will help to reveal individuals with increased risk of osteoporosis and suggest a personalized clinical approach to prevent or at least to delay the development of this pathology. The prevalence of osteoporosis is different in various ethnicities (2). Evaluation of genetic predisposition to osteoporosis is especially important, because this disease is asymptomatic; the first clinical manifestation in the majority of cases is low energy fractures, and the number of older people, having elevated risk of fragility fractures, is increasing. Postmenopausal women lose important bone protectors (estrogens), and their bone resorption rate increases dramatically (3).

The human *VDR* gene is located on the short arm of chromosome 12. It consists of 9 exons and encodes a 427 amino acid protein (4). In the *VDR* gene, several polymorphic sequence variations have been reported, which can occur in coding or noncoding parts of the gene and lead to changes in the protein sequence or affect the degree of gene expression (5). These include single nucleotide polymorphisms (SNP) that can be identified with the appropriate restriction endonuclease enzymes, such as ApaI (rs7975232), BsmI (rs1544410), FokI (rs2228570), TaqI (rs731236), and Cdx2 (rs11568820).

*VDR* ApaI (rs7975232) gene polymorphism is located in the 3′-regulatory region of *VDR* gene (in intron 8). There is no functional effect of this variant described, although some authors suggested its effect on mRNA stability (5). It was found that BsmI (rs1544410) variant is significantly associated with the increased risk of developing PMO (6) and antiresorbtive treatment responses (7). *VDR* TaqI (rs731236) gene polymorphism is located in exon 9 and it has been proved to affect mRNA stability, influencing biological function of vitamin D (8). These three SNPs are located at the 3′-terminus of *VDR* gene and frequently reported to be in linkage disequilibrium (LD). The FokI (rs2228570) polymorphism is located in the second exon of the *VDR* gene, plays an important role in post transcriptional processes and causes the production of two different VDR protein variants: long and short variants (5). Compared with the long VDR form, the short form has greater transcriptional activation capability. In meta-analysis, *VDR* FokI polymorphism was significantly associated with higher risk of developing PMO in Asian, but not in Caucasian populations (6). Cdx2 variant is located in the 5′-promoter region of the *VDR* gene. The *VDR* Cdx2 G-variant reduces transcriptional activity of the gene to 70% of the A allele (9).

In recent years, multiple studies have been performed to investigate correlation between *VDR* gene variants and osteoporosis risk, suggesting the presence of ethnic differences in the genetic association with osteoporosis. But there is still no clear evidence about their effects in performed recent meta-analyses (6, 10–12). Therefore, to improve the significance of associations, it is necessary to perform evaluation of combinations of genetic variants with established effects on independent cohort.

The aim of this study was to compare the associations of selected polymorphic variants within *VDR* gene with the risk of osteoporosis in Belarusian and Lithuanian postmenopausal women.

## Materials and Methods

### Subjects and Clinical Assessment

Based on a case–control design, 149 patients with postmenopausal osteoporosis (PMO group) and 172 asymptomatic controls (CON group) participated in the study. One hundred twenty-one Belarusian patients and 127 controls were recruited from Minsk City Center for osteoporosis and bone-muscular diseases prevention (Minsk, Belarus), while 28 Lithuanian patients and 45 controls were recruited from National Osteoporosis Center (Vilnius, Lithuania). All subjects signed written informed consent after being fully informed about the nature of the study according to Helsinki Declaration of 1975, as revised in 2000. Local Research Ethics Committee at the Belarusian Medical Academy of Postgraduate Education and Lithuanian Regional Biomedical Research Ethics Committee approved the study protocol. The exclusion criteria for both groups were chronic diseases, oncology, hypercalcemia, and use of glucocorticosteroids. The women with the clinical diagnosis of osteoporosis (the BMD *T*-score of −2.5 or lower at the femoral neck or the lumbar spine) and at least 2 years postmenopausal were defined as the patients with PMO. The control group comprised postmenopausal women with BMD *T*-score of >−2.5 and without previous fragility fractures. The data of the medical history and the fracture history were obtained by a clinical expert.

### BMD Measurement

Bone mineral density was measured at the lumbar spine and both proximal femurs using dual-energy X-ray absorptiometry (Prodigy, GE Lunar, Madisson, WI, USA). The lowest value from right or left femur and lumbar spine L_1_–L_4_ BMD was taken and used in further comparative analysis.

### Genotyping

For genetic analyses, venous blood samples were taken from the cubital vein using the Vacutainer system with EDTA (Beckton-Dickinson, Franklin Lakes, NJ, USA). DNA was isolated from bloodspots dried on special NucleoSafe cards (Macherey-Nagel, Germany) using the standard proteinase K digestion, phenol–chloroform extraction, and ethanol precipitation. The DNA solution was extracted with a phenol–chloroform–isoamyl alcohol mixture to remove protein contaminants and then was precipitated with 100% ethanol. The DNA was pelleted after the precipitation step, washed with 70% ethanol to remove salts and small organic molecules, and resuspended in a buffer at a concentration suitable for further investigation (20–120 ng/µL). The quality and purity of DNA samples were checked using Qubit 2 Fluorimeter (Thermo Fisher Scientific, USA).

Selected polymorphic variants (ApaI rs7975232, BsmI rs1544410, TaqI rs731236, and Cdx2 rs11568820) in *VDR* gene were determined using the polymerase chain reaction and restriction fragment length polymorphism (PCR-RFLP) analysis as described earlier (13). Briefly, the PCR reaction system consisted of 10-µL 10 × PCR buffer (1 × buffer = 10 mM Tris–HCl, pH 8.3; 50 mM KCl; 1.25 mM MgCl_2_), 1.0 µL of 10 × dNTPs (0.2 mM), 1.0 µL of each primer, 0.5 µL of polymerase, 3.5 µL of mQ water, and 10 ng of genomic DNA. The PCR was performed with an initial denaturation at 95°C for 15 min, followed by 28 cycles of denaturation at 99°C for 1 s, annealing at 60°C for 10 s, and extension at 72°C for 10 s. The PCR amplification was carried out in an automated thermal cycler (C1000, Bio-Rad, USA). The final extension was performed at 72°C for 1 min. The PCR products were size-separated by electrophoresis on the 10% polyacrylamide gel at 125 V for 1 h. The 100-bp DNA ladder (Thermo Fisher Scientific, Lithuania) was used to determine the fragments size.

### Statistical Analysis

Kolmogorov–Smirnov test was used to assess the normality of data distribution. Normally distributed data are presented as mean and compared using Student’s *t*-test. The data which are not normally distributed are presented as median (25, 75% interquartile range) and compared using Mann–Whitney *U*-test.

Based on the determined frequencies of genotypes and by using the Pearson chi-square (χ^2^) test, the Hardy–Weinberg equilibrium was assessed. Crude odds ratios (OR) were reported with 95% confidence intervals (95% CI) and calculated in comparison to reference (wild-type) genotype. Logistic regression models were used to assess difference between the characteristics of PMO and CON groups for categorical data and for comparison of allele, genotype, and haplotype frequencies between these groups. The statistical analysis was performed using the freely available programming language R (http://r-project.org) with package “SNPassoc” (version 1.9-2). LD between the genetic variants was determined using “haplo.stats” R-package. The differences between the groups were considered statistically significant at *p* < 0.05.

## Results

### Participant Characteristics

Each subject was supplied with questionnaire, containing personal information and variables, necessary for the study. The participants within the PMO and CON groups were matched for age, height, sex, and BMI—no statistically significant differences were found (Table 1). Both groups included Belarusian and Lithuanian individuals—postmenopausal women of the same age. The comparison of Belarusian and Lithuanian groups has not revealed any significant differences in alleles and genotypes distribution. The CON group had significantly higher spine and femur neck BMD level compared to PMO group (*p* < 0.01).

**Table 1:** Clinical characteristics of analyzed patients with postmenopausal osteoporosis (PMO) and control (CON) groups.

	PMO (*n* = 149)	CON (*n* = 172)	*p-*Value
Age, years	61.4 (6.5)^a^	57.5 (7.3)^a^	>0.05
Height, cm	159.2 (8.3)^a^	165.1 (5.8)^a^	>0.05
Weight, kg	64.5 (7.2)^a^	73.3 (5.1)^a^	>0.05
Body mass index, kg/m^2^	26.7 (3.2)^a^	27.3 (4.7)^a^	>0.05
Years after menopause	11.3 (4.5)^a^	8.2 (2.7)^a^	>0.05
Spine bone mineral density (BMD), g/cm^2^	0.944 (0.831; 1.090)^b^	1.152 (1.024; 1.240)^b^	<0.01
Femoral neck BMD, g/cm^2^	0.803 (0.707; 0.914)^b^	0.983 (0.913; 1.13)^b^	<0.01

*^a^SD*.

*^b^IQR, 25–75% interquartile range*.

### Genotype and Allele Frequencies Distribution of Single *VDR* Gene Variants

Four *VDR* gene restriction fragment length polymorphisms (ApaI, BsmI, TaqI, and Cdx2) were selected following the literature review for consideration in Caucasian population (5, 6, 14, 15). Search of risk alleles and assessment of their combined action in present study was performed on the independent cohort. The genotype and allele frequencies of the analyzed *VDR* gene single SNPs are presented in Table 2. The distribution of all four analyzed gene polymorphisms in controls and in patients with postmenopausal osteoporosis was in correspondence with the one expected from the Hardy–Weinberg equilibrium (*p* > 0.05 in all cases).

**Table 2:** The genotype and allele frequencies (in %) of *VDR* gene variants.

Gene variant	Genotype, major allele	PMO, *n* = 149	Control, *n* = 172	OR (95% CI)	*p-*Value
*VDR* ApaI rs7975232	AA	18.1	34.9	1.0	
	AC	45.0	43.0	1.1 (0.7–1.7)	**0.008**
	CC	36.9	22.1	**2.1 (1.3–3.4)**	
	A allele	40.6	56.4	**0.5 (0.4–0.7)**	**0.0007**

HWE *p*-value		0.41	0.12		

*VDR* BsmI rs1544410	AA	21.8	37.2	1.0	
	AG	42.9	42.4	1.7 (1.0–3.0)	**0.002**
	GG	35.4	20.4	**3.0 (1.6–5.4)**	
	A allele	43.2	58.4	**0.5 (0.4–0.7)**	**0.0001**

HWE *p*-value		0.13	0.12		

*VDR* TaqI rs731236	TT	25.5	33.7	1.0	
	TC	41.6	43.0	1.3 (0.8–2.2)	0.1
	CC	32.9	23.3	1.9 (1.0–3.0)	
	T allele	46.3	55.2	**0.7 (0.5–0.95)**	**0.02**

HWE *p*-value		0.05	0.09		

*VDR* Cdx2 rs11568820	GG	68.4	71.6	1.0	
	GA	30.1	27.6	1.3 (0.7–2.2)	0.15
	AA	1.5	0.8	–	
	G allele	83.5	85.4	0.9 (0.5–1.4)	0.52

HWE *p*-value		0.28	0.2		

There was a statistically significant difference in genotype and allele distribution for *VDR* ApaI, BsmI, and TaqI gene variants revealed between PMO and CON groups (Table 2). The *CC* genotype of *VDR* ApaI was significantly over-represented in PMO (36.9%) group when compared to the CON (22.1%) group (OR = 2.1, 95% CI 1.3–3.4, *p* = 0.008). The CON group individuals were more likely to carry *VDR* ApaI A allele (56.4%), compared to the PMO group (40.6%, OR = 0.5, 95% CI 0.4–0.7, *p* = 0.0007). The differences in the genotype (*p* = 0.002) and allele (*p* = 0.0001) frequency distributions of the *VDR* BsmI gene variant between the PMO and CON groups were also significant. More specifically, the risk of PMO was significantly higher for the bearers of GG-genotype compared to reference wild-type AA-genotype (OR = 3.0, 95% CI 1.6–5.4, *p* = 0.002).

There was, however, no statistically significant difference in *VDR* Taq genotype distribution (*p* = 0.1), though the T allele was over-represented in the asymptomatic CON (55.2%) group compared to the PMO (46.3%) group (OR = 0.7, 95% CI 0.5–0.95) and the C-allele was over-represented in the PMO (53.7%) group compared to the CON (44.8%) group (OR = 1.4, 95% CI 1.1–2.0, *p* = 0.02 in both cases). No significant differences in the *VDR* Cdx2 genotype distribution (*p* = 0.15) and allele frequencies (*p* = 0.52) between the PMO and CON groups were observed.

### LD Analysis of *VDR* Gene

**Figure 1:**
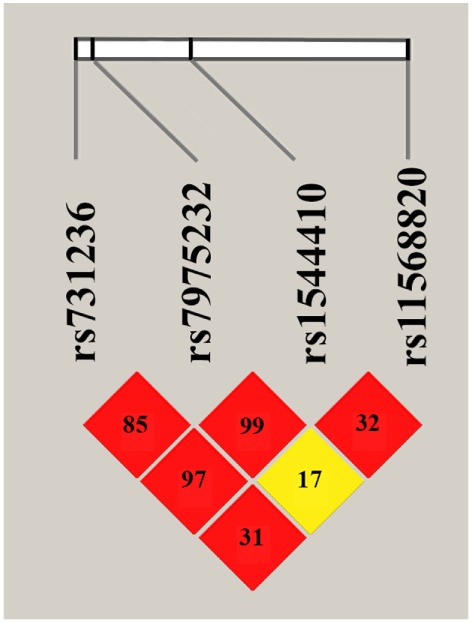
Linkage disequilibrium plot for *VDR* ApaI, BsmI, TaqI, and Cdx2 variants. The LD plot was build using combined genotype data from postmenopausal osteoporosis and control groups (constructed with “haplo.stats” package for R). LD is displayed as pairwise *D*’ values multiplied by 100 and given for each single nucleotide polymorphism combination within each cell. Red cells correspond to *p* < 0.0001, yellow—*p* > 0.1.

The results of LD analysis are presented in the Figure 1. LD plot was constructed using combined genotype data from PMO and CON groups. The three SNPs, ApaI (A/C), BsmI (A/G), and TaqI (T/C) of *VDR* gene are in a very strong LD (the measure *D’* is very close to 1, *p* = 0.01). The positive coefficient of correlation *r* suggests that major alleles of *VDR* ApaI, BsmI, and TaqI gene variants are likely to be inherited together, as well as minor alleles.

No significant LD was found between *VDR* Cdx2 and other three analyzed gene variants. Thus, two haplotypes A-A-T and C-G-C are inferred with high probability from revealed *VDR* polymorphisms with high *D*’ measure, allowing further complex analysis of allelic combinations.

### Haplotype Analysis

**Figure 2:**
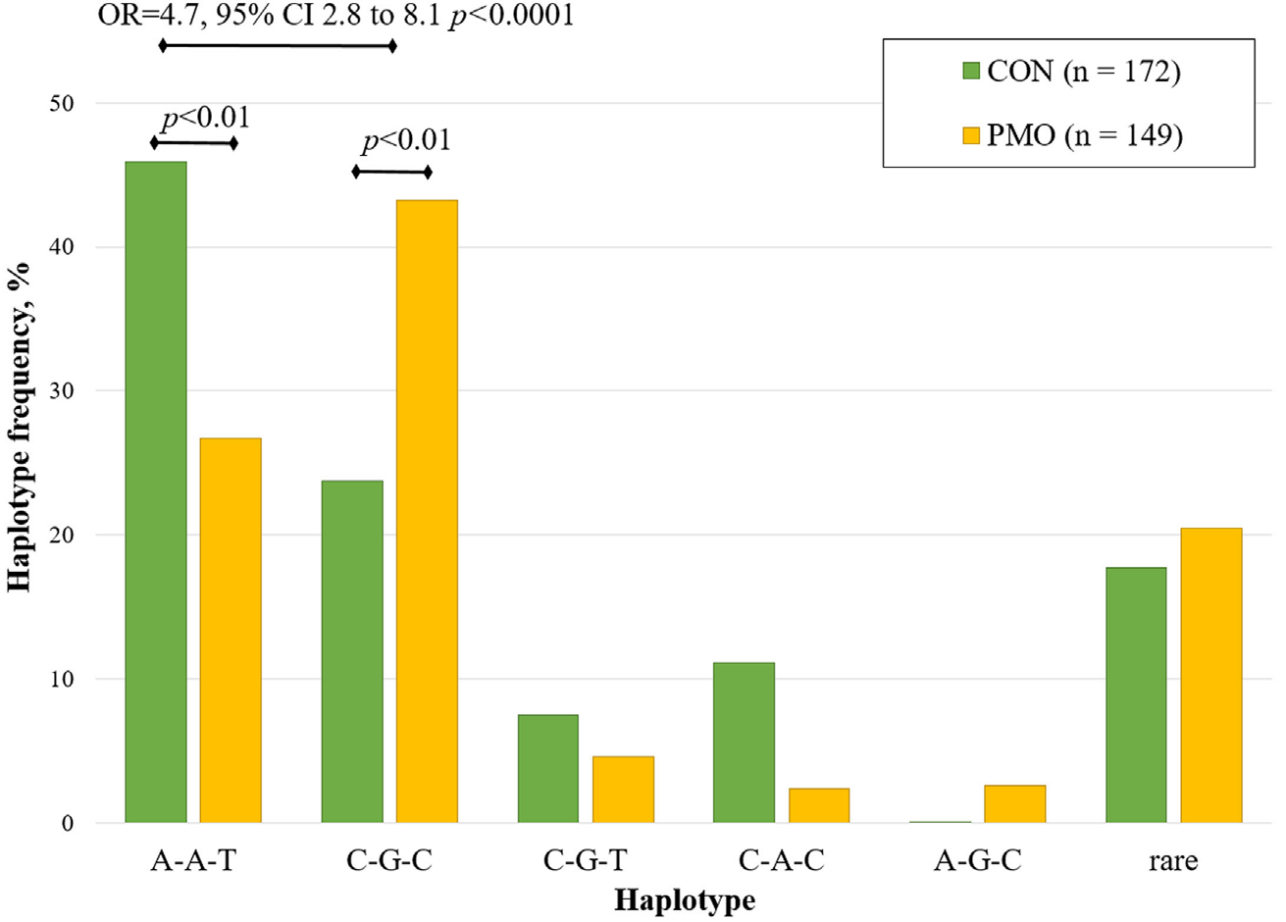
Estimated haplotype frequency distribution of *VDR* ApaI, BsmI, and TaqI variants in the postmenopausal osteoporosis (PMO) and control (CON) groups.

The haplotype analysis was performed for three *VDR* gene polymorphisms: ApaI, BsmI, and TaqI (Figure 2). Haplotypes were constructed from all possible allelic combinations of three *VDR* polymorphisms and compared between the PMO and CON groups.

Five haplotypes (A-A-T, C-G-C, C-A-C, A-G-C, and C-G-T) of the possible eight combinations were inferred at a frequency of greater than 5%. The A-A-T haplotype was the most frequent haplotype (total frequency 36.8%), constructed from three wild-type allele variants. This haplotype was significantly over-represented in CON group (46.0%) compared to the PMO group (26.7%, *p* < 0.01). The total frequency of C-G-C haplotype was 32.4%, it was significantly under-represented in CON group (23.7%) compared to PMO group (43.2%, *p* < 0.01). For the bearers of C-G-C haplotype, the risk of PMO was significantly higher compared to the reference (wild-type) haplotype (OR = 4.7, 95% CI 2.8–8.1, *p* < 0.0001). None significant association was found for other revealed haplotypes.

## Discussion

This case–control study was performed to investigate the role of *VDR* gene in PMO in combined cohorts of Caucasian (Belarusian and Lithuanian) women. Because PMO is a multifactorial disorder, genetic variants within the *VDR* gene, which is an important regulator of bone remodeling, may be a contributing risk factor. For the best of our knowledge, this is the first study demonstrating an association of four *VDR* variants with PMO risk in cohort of Belarusian and Lithuanian patients.

*VDR* gene variants were selected from key publications with established associations with PMO (5, 6, 14, 15) and analyzed in independent population for assessing their combined action. A total of four polymorphic variants of *VDR* gene were selected and evaluated for this study. These variants are established as PMO risk factors, so we are mainly interested to know if their effect is similar to other populations or may be different and what will be their combined action.

The cases and controls involved in the analysis were well defined with similar inclusion criteria. The descriptive analysis of our random selection of subjects showed that PMO and CON groups were matched for age, height, weight, sex, and BMI. Spine and femoral BMD levels were significantly different between PMO and CON groups. The observed genotype frequencies in CON group did not deviate from Hardy–Weinberg equilibrium (*p* > 0.05 in all cases), while in PMO group it deviated (*VDR* Taq, *p* = 0.05). We also revealed significant differences in *VDR* polymorphic alleles (ApaI, BsmI, and TaqI) and genotypes (ApaI and BsmI) frequency distribution between PMO and CON groups.

Obtained genotype and allele frequencies in CON group (Table 2) for *VDR* ApaI were close to those of Caucasian subjects reported in HapMap (16), for *VDR* BsmI—in British (17), Spanish (18), and Slovenian (19) populations, *VDR* TaqI—in Czech population (20), *VDR* Cdx2—in Slovenian population (19). The contrast with the data reported in other Caucasian studies (6) is likely related to both ethnic differences and differences in inclusion criteria.

The main finding of single *VDR* gene variants association analysis is that the presence of *VDR* ApaI CC and BsmI GG homozygous genotypes is significantly associated with increased PMO risk, being over-represented in PMO group. By comparison of *VDR* TaqI allele frequencies in CON and PMO groups, we found that allele C also represented a risk factor (OR = 1.4, *p* = 0.02). This data are in accordance with the data reported in meta-analysis (6), where ApaI and BsmI were the most frequent markers, associated with PMO risk. Currently it is accepted that *VDR* BsmI polymorphism is related to BMD level, but its effect is relatively small and strongly influenced by external factors like diet (1). Unlike *VDR* BsmI, ApaI, and TaqI polymorphisms affect mRNA stability, which results in change of biological functions of vitamin D (6, 21).

The absence of significant association for *VDR* Cdx2 may be explained by very low number of individuals with AA-genotypes (less than five individuals in each subgroup).

As shown in Figure 1, three of four investigated variants were in strong LD. This finding is in agreement with previous studies that have found strong linkage between ApaI, BsmI, and TaqI variants of *VDR* gene located within the chromosomal region 12q12. The greatest degree of LD was found between ApaI and BsmI, followed by BsmI and TaqI, and then ApaI and TaqI. The same trend was reported in the meta-study for Caucasian populations (22), as well as for British population (23), opposite trend observed in Italian population (24). Most likely, the strong LD coefficient may be explained by the location of all three markers in the ninth exon of *VDR* gene, as well as by the adaptive advantage of a particular allelic combination. The absence of LD between Cdx2 (located in the promoter region of *VDR* gene) and other three variants is in good accordance with the literature (23).

Positive correlation between *VDR* ApaI, BsmI, and TaqI variants suggests that their major alleles are associated together, making them likely to be inherited jointly. Using logistic regression, we have analyzed the global distribution of all revealed haplotypes in PMO and CON groups. Inferred haplotypes of ApaI, BsmI, and TaqI variants were significantly associated with PMO risk (global haplotype association *p* < 0.0001).

The most frequent haplotype was wild-type A-A-T (36.8%) followed by C-G-C (32.4%). These haplotypes were more common than other haplotypes in CON and PMO groups. Very close haplotype frequency distribution was reported for Caucasian (22), Dutch (25), and Italian (24) women. By excluding too rare haplotypes, most common were compared with reference haplotype A-A-T. The data show that for the bearers of non-favorable haplotype C-G-C, the risk of PMO is significantly higher. No protective variants were revealed in this study.

It is necessary to mention that, in contrast to ApaI and BsmI, *VDR* TaqI variant is constituted by the silent replacement of thymine (T) by cytosine (C) and does not change the amino acid sequence of VDR protein (5). This evidence partly contradicts with the result of this study, where significant association of PMO risk with single *VDR* TaqI allele variants or *VDR* ApaI-BsmI-TaqI haplotypes was found. These three SNPs are located at the 3′ end of the *VDR* gene and are at very high degree of LD. Therefore, with high probability the risk allele of TaqI variant is inherited together with risk alleles of ApaI and BsmI variants, and *vice versa*. For this reason, the effect of *VDR* TaqI variant can be explained by strong LD, when they are found in specific haplotypes, and due to the effects of ApaI and BsmI polymorphisms on PMO risk. Another possible explanation of *VDR* TaqI significant association with PMO risk are epigenetic (methylation) processes (26).

Considering the contrasting results in different studies of the genetic predisposition to osteoporosis, it seems that the best interpretations are possible if a complex of genetic variants and haplotypes is evaluated. In addition, it is very important to analyze the association of established risk variants on independent cohort, which will help to escape risk score summarization paradox. The present study is the first such research, performed on independent cohorts for evaluation of combined effect of different genetic variants with known associations, as well as to assess if the overall pattern of association is similar to other populations. Moreover, basic studies will help to reveal a deeper knowledge of the molecular mechanisms of bone disorders regulated by vitamin D through its receptors.

The limitations of current study include a low number of Lithuanian participants, which is not sufficient for comparison of *VDR* variants genotype distribution between Lithuanian and Belarusian populations. Also, the sample size was not enough big to perform the adjustments for additional covariates (such as BMI, height, weight, vitamin D level, fractures incidence). Further, well-designed studies with larger sample sizes of both ethnic populations will clarify data, obtained in present studies.

Thus, the most important result of this study is that for the women-bearers of C-G-C haplotype, there is a 4.7-fold risk of developing PMO. Our study suggests that variants of *VDR* gene are associated with the risk of PMO, although different polymorphisms might have different influences. The overall pattern of known *VDR* markers of PMO risk in combined Belarusian and Lithuanian population is close to other Caucasian populations. Further work will also include analysis of interaction between *VDR* variants and other genetic markers, playing important roles in genetic predisposition to osteoporosis.

## Data Availability Statement

The raw data supporting the conclusions of this manuscript will be made available by the authors, without undue reservation, to any qualified researcher.

## Author Contributions

PM collected the data, performed statistical analysis, and drafted the research paper. ER, IM, MT, and VA supervised and guided the design and analysis of the research project. AR and VS collected patient sample and performed clinical survey of Belarusian patients. MT collected patient sample and performed clinical survey of Lithuanian patients. KK performed DNA extraction and genotyping. All authors were involved in drafting and revising the manuscript.

## Conflict of Interest Statement

The authors declare that the research was conducted in the absence of any commercial or financial relationships that could be construed as a potential conflict of interest.
